# Diagnostic Value of Point-of-Care Ultrasound for Sarcopenia in Geriatric Patients Hospitalized for Hip Fracture

**DOI:** 10.3390/jcm14155424

**Published:** 2025-08-01

**Authors:** Laure Mondo, Chloé Louis, Hinda Saboul, Laetitia Beernaert, Sandra De Breucker

**Affiliations:** 1 Geriatric Department, Hôpital Universitaire de Bruxelles (HUB), Erasme Campus, 808 Route de Lennik, 1070 Brussels, Belgium; laure.mondo@ulb.be (L.M.);; 2 Geriatric Department, Hôpitaux Universitaires de Genève (HUG), Rue Gabrielle Perret-Gentil 4, 1205 Geneva, Switzerland

**Keywords:** sarcopenia, hip fracture, ultrasound, rectus femoris

## Abstract

**Introduction**: Sarcopenia is a systemic condition linked to increased morbidity and mortality in older adults. Point-of-Care Ultrasound (POCUS) offers a rapid, bedside method to assess muscle mass. This study evaluates the diagnostic accuracy of POCUS compared to Dual-energy X-ray Absorptiometry (DXA), the gold standard method, and explores its prognostic value in old patients undergoing surgery for hip fractures. **Patients and Methods**: In this prospective, single-center study, 126 patients aged ≥ 70 years and hospitalized with hip fractures were included. Sarcopenia was defined according to the revised 2018 EWGSOP2 criteria. Muscle mass was assessed by the Appendicular Skeletal Muscle Mass Index (ASMI) using DXA and by the thickness of the rectus femoris (RF) muscle using POCUS. **Results**: Of the 126 included patients, 52 had both DXA and POCUS assessments, and 43% of them met the diagnostic criteria for sarcopenia or severe sarcopenia. RF muscle thickness measured by POCUS was significantly associated with ASMI (R^2^ = 0.30; *p* < 0.001). POCUS showed a fair diagnostic accuracy in women (AUC 0.652) and an excellent accuracy in men (AUC 0.905). Optimal diagnostic thresholds according to Youden’s index were 5.7 mm for women and 9.3 mm for men. Neither RF thickness, ASMI, nor sarcopenia status predicted mortality or major postoperative complications. **Conclusions**: POCUS is a promising, accessible tool for diagnosing sarcopenia in old adults with hip fractures. Nonetheless, its prognostic utility remains uncertain and should be further evaluated in long-term studies.

## 1. Introduction

Sarcopenia is defined by a progressive decline in skeletal muscle mass and function that occurs with aging. This process may begin as early as the age of 40 and is termed primary sarcopenia. Muscle loss can also result from external factors such as physical inactivity, malnutrition, or chronic illnesses and is then classified as secondary sarcopenia [1]. Estimating the global prevalence of sarcopenia is challenging due to the coexistence of multiple consensus definitions and anthropometric variations across different continents, which then influence diagnostic thresholds. Nevertheless, it is estimated to affect between 10% and 27% of adults over the age of 60 worldwide [2]. Sarcopenia represents a major public health issue, as it is associated with an increased risk of morbidity, mortality, hospitalization, functional decline, falls, and fractures [3]. In geriatric patients hospitalized with hip fractures, the reported prevalence ranges from 44% to 76.2%, depending on the study [4,5].

Hip fracture is a common and serious condition in older adults, with one-year mortality rates ranging from 16.6% to 31% [6,7]. Several studies have identified prognostic factors, including advanced age, male sex, high levels of dependency, comorbidities (cardiovascular, pulmonary, or cancer), delayed surgical management, and preoperative anemia [6,7,8]. Sarcopenia has also been identified as a long-term prognostic factor in hip fractures, the 7-year mortality rate being 1.7 times higher in sarcopenic patients compared to controls [9].

Among the existing definitions of sarcopenia, the European Working Group on Sarcopenia in Older People (EWGSOP) first proposed a definition in 2010, which was then revised in 2018, following a three-step diagnostic algorithm. The diagnosis is confirmed when low muscle strength is associated with low muscle mass, with or without low physical performance [10].

Dual X-Ray Absorptiometry (DXA) is considered one of the standard reference methods for evaluating muscle mass, in both clinical and research settings. It provides a comprehensive body composition analysis, with lower radiation exposure and reduced cost compared to other imaging modalities such as computed tomography (CT) or magnetic resonance imaging (MRI) [10,11]. Ultrasound imaging has recently emerged as a promising alternative for muscle assessment, as it can be performed at the patient’s bedside, is non-irradiating, cost-effective, and allows for both quantitative and qualitative evaluation [12]. Compared with traditional imaging methods (DXA, CT, and MRI), ultrasound has demonstrated good reliability and validity for assessing muscle mass in community-dwelling elderly adults and has shown satisfactory correlation with DXA [13,14]. Despite its potential, ultrasound is yet to be included in current diagnostic guidelines due to the absence of consensus on protocols and normative and diagnostic cut-off values [12]. More recently, portable ultrasound devices (Point-of-Care Ultrasound, POCUS) have allowed trained clinicians to perform bedside assessments for screening, diagnosis, and monitoring and assisting with interventions, which is particularly advantageous in geriatric care, where patient mobility is often limited [15]. POCUS is more affordable and user-friendly than standard ultrasound systems, although it still requires specific training and does remain operator-dependent [12]. POCUS has shown a strong correlation with the total skeletal muscle cross-sectional area at the L3 vertebral level when compared to CT imaging in cancer patients undergoing abdominal surgery and with BIA and anthropometric measures in ambulatory elderly adults [16,17]. However, to date, no studies have evaluated the use of POCUS to assess muscle mass in patients admitted with hip fractures. In parallel, POCUS has also shown promise in other applications such as periosteal and fracture assessment, further supporting its potential as a versatile bedside tool in orthogeriatric care [18].

The primary aim of our study was to assess the diagnostic accuracy of POCUS in evaluating muscle mass among patients aged 70 and older and hospitalized with hip fractures, using rectus femoris muscle thickness as a surrogate marker and comparing it to the Appendicular Skeletal Muscle Mass Index (ASMI) measured by DXA. The secondary objective was to examine the prognostic value of rectus femoris thickness (as assessed by POCUS), of ASMI (as measured by DXA), and of sarcopenia diagnosis according to the EWGSOP2 criteria, in relation to severe postoperative complications and mortality.

## 2. Materials and Methods

### 2.1. Study Design

We conducted a prospective, single-center study conducted between 1 April 2022, and 1 December 2024, in a geriatric ward in Brussels, Belgium.

### 2.2. Inclusion and Exclusion Criteria

All patients aged 70 and older and surgically treated for a hip fracture were included, except those who declined participation. Patients with confirmed pathological fractures, periprosthetic fractures, or fractures resulting from high-velocity trauma were excluded. Included patients, or their legal representatives, received information about the study by letter. The study was approved by the Ethics Committee of Erasme Hospital (CCB B4062022000132; date of approval: January 2022).

### 2.3. Data Analysis

The following information and data were collected within the 7 days following admission:

- Socio-demographic, clinical, and geriatric assessment data: age, gender, living situation prior to admission (home or nursing home), body mass index, comorbidities (hypertension, diabetes, heart failure, valvular disease, chronic kidney disease, chronic obstructive pulmonary disease, or history of cerebrovascular disease), polypharmacy (use of >5 medications), basic activities of daily living (ADL), instrumental activities of daily living (IADL), nutritional status (MNA-sf score), comorbidity (CIRS-G score), cognitive impairment, and depression.
- Surgical data: type of fracture (intra- or extracapsular), type of surgical procedure (intramedullary nail, hemiarthroplasty, total hip replacement, triple screw fixation, or plate and screws), delay between admission and surgery (hours), and surgery duration (minutes).
- Biological parameters at admission: hemoglobin, white blood cell count, CRP, albumin, and Vitamin D levels.
- Postoperative complications: the Clavien–Dindo classification was used within 72 h, and the scores were dichotomized into non-severe (grades 1 and 2) and severe (grades 3 to 5) complications [19,20]. Inpatient falls, delirium (detected by at least one positive CAM—Confusion Assessment Method—score), administration of red blood cell transfusions, pressure injuries, and length of stay were also recorded. Inpatient mortality rates were assessed, as well as at 1 month, 3 months, and 6 months post-discharge via electronic health records or contact with the patient’s General Practitioner.
- Sarcopenia assessment: Sarcopenia was defined according to the revised EWGSOP2 criteria [6]. Probable sarcopenia was considered if the maximal grip value of the dominant hand was inferior to 27 kg in men and 16 kg in women. Handgrip strength (HGS) was assessed before surgery when possible, or far from the time of surgery, by two trials of the dominant hand using a standard hand-held dynamometer (JAMAR; TEC; Clifton, NJ, USA), and the maximum value was retained. Sarcopenia was diagnosed as a low grip strength associated with a low muscle mass index. ASMI was measured by DXA, and a low muscle mass was determined by using the cut-off of 5.5 kg/m^2^ for women and 7.0 kg/m^2^ for men. Sarcopenia severity was assessed via the mobility component of the MNA-SF, due to the inability of patients to perform physical performance tests postoperatively. This component is considered as a valid to predict gait speed in old, hospitalized patients [21]. The EWGSOP2 categories were dichotomized into two groups: the non-sarcopenic control group including patients with no sarcopenia and probable sarcopenia and the sarcopenia group including confirmed sarcopenia and severe sarcopenia. Rectus femoris thickness was measured using a portable ultrasound device (Clarius PA HD3 Wireless, Belfast, Ireland), with the patient in a supine position, halfway between the anterior superior iliac spine and the superior border of the patella, on the non-fractured leg with the knee extended and relaxed, as according to the recommendations [22]. Measurements were performed by two trained ultrasound operators. Inter-operator reliability was analyzed prior to the study and showed an intraclass correlation coefficient (ICC) between 0.964 and 0.997 (*n* = 9).

### 2.4. Statistical Analysis

Statistical analyses were performed by STATA SE, version 18.0 (Stata Corp, College Station, TX, USA, 2023). The normality of the distribution of continuous variables were checked with the Shapiro–Francia tests for normality. Normally distributed variables were expressed as means ± standard deviation, non-parametric continuous variables were expressed as medians [25–75 interquartile range], and logistic, ordinal, and nominal variables were expressed in numbers (percentages).

Linear regression was used to assess the association between ASMI and rectus femoris thickness. ROC curves were generated to compare POCUS-based rectus femoris thickness with the standard reference DXA using EWGSOP2 ASMI thresholds. The area under the curve (AUC) was calculated, and the optimal threshold was defined using Youden’s index (sensitivity + specificity − 1).

The prognostic values of rectus femoris thickness, ASMI, and sarcopenia diagnosis for severe postoperative complications (Clavien–Dindo score > 2) and for inpatient and 1,3, and 6 months post-discharge mortality were evaluated using univariate logistic regression. Multivariate analysis was not feasible due to the low number of events.

A *p*-value < 0.05 was considered statistically significant.

## 3. Results

**Figure 1:**
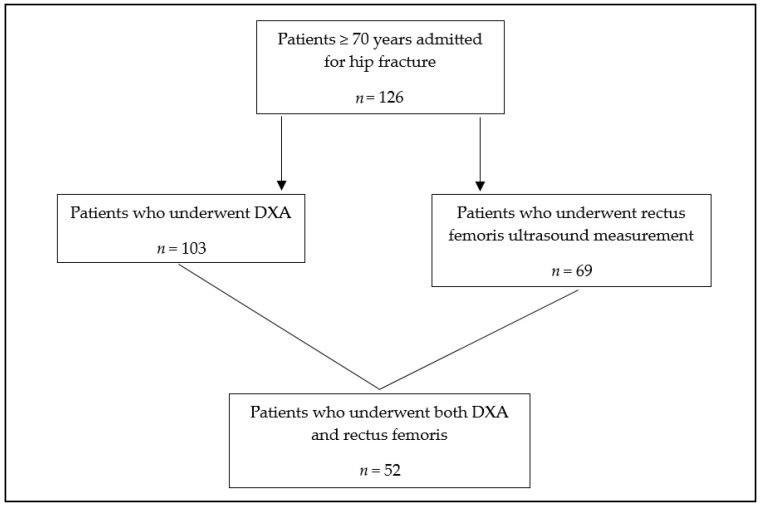
Patient selection flowchart.

A total of 126 patients aged over 70 years and hospitalized for a hip fracture were included in the study. Among them, 103 underwent muscle mass measurement by DXA, 69 underwent ultrasound measurement of the rectus femoris, and 52 underwent both evaluations (Figure 1).

### 3.1. Descriptive Analysis

Table 1 summarizes the patient characteristics. The mean age was 85.3 ± 6.6 years, and 72.2% were women. There was a high prevalence of malnutrition, cognitive impairment, and polypharmacy. Most patients had either an intracapsular hip fracture (51.6%) or an intertrochanteric fracture (42.8%).

The characteristics relating to sarcopenia are summarized in Table 2. In our sample, 43% of patients had sarcopenia, including 35.5% of women and 66.7% of men. Among the total sample, 11.9% had severe sarcopenia.

### 3.2. Analysis of the Association Between Rectus Femoris Thickness by POCUS and ASMI by DXA

The linear regression analysis between rectus femoris thickness and ASMI showed a significant positive association (R^2^ = 0.30; *p* < 0.001).

**Figure 2:**
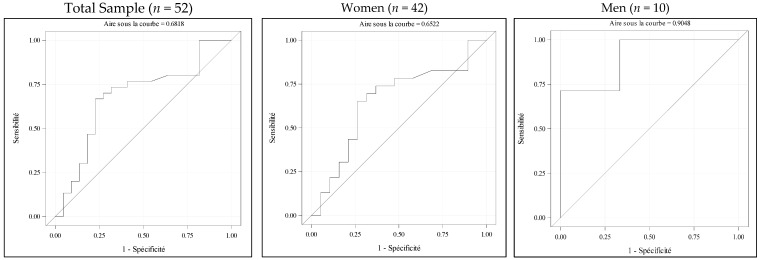
Analysis of the diagnostic performance of ultrasound-based rectus femoris thickness measurement for the identification of sarcopenia.

The ROC curves analysis showed that rectus femoris thickness was an acceptable predictor of low muscle mass compared to DXA in the overall sample, and in the female subgroup, with an AUC of 0.687 (sensitivity of 66.7% and specificity of 77.3%) and 0.652 (sensitivity of 65.2% and specificity of 73.9%), respectively. The performance was excellent in men, with an AUC of 0.905 (sensitivity of 71.4% and specificity of 100%) (Figure 2).

The threshold values of rectus femoris thickness used to identify sarcopenic patients, determined using Youden’s index, were 6.1 mm in the overall population, 5.7 mm in women, and 9.3 mm in men.

### 3.3. Analysis of Factors Associated with Severe Complications and Mortality

Sarcopenia-related data associated with severe complications are presented in Table 3. None of the sarcopenia-related measures were predictive of severe complications. The univariate logistic regression analysis of other factors associated with mortality are provided as Supplementary Material for reference.

Furthermore, none of the sarcopenia measures were predictive of mortality, in-hospital or at 1, 3, or 6 months. (Table 4).

All collected variables examined in univariate analyses for their association with 6-month mortality and severe postoperative complications (Clavien > 2) are detailed in Supplementary Table S1 and Supplementary Table S2, respectively. Six-month mortality was mainly associated with older age, male sex, cardiac or renal comorbidities, and postoperative complications. Severe postoperative complications were associated with renal and valvular diseases, surgical delay, and inflammatory status at admission.

## 4. Discussion

The primary objective was to evaluate the diagnostic validity of POCUS ultrasound to assess muscle mass in geriatric patients admitted with hip fractures. The rectus femoris thickness is a superficial muscle, easily accessible by ultrasound. It is considered a good indicator of total muscle mass in healthy older adults [23,24]. Moreover, the anterior thigh muscles are known to be amongst the first affected by age-related muscle wasting, making them a pertinent target for early sarcopenia diagnosis [12].

To the best of our knowledge, this is the first study validating this measurement method in hospitalized elderly patients with hip fractures, using ASMI by DXA as a comparator. Only one prior study was conducted in a similar population, showing good correlation between quadriceps thickness (combining rectus femoris and vastus intermedius) via conventional ultrasound and SMI measured by BIA [14]. This is also the first study using Point-of-Care Ultrasound for this indication—although rectus femoris thickness has already been validated as a muscle mass assessment parameter via conventional ultrasound in old community-dwelling people, having been compared to both DXA [23] and BIA [25].

Our study showed that POCUS had a good diagnostic performance in evaluating muscle mass, with satisfactory results in women and excellent results in men, supporting its probable effectiveness in identifying sarcopenic patients with hip fractures. We proposed diagnostic thresholds with sensitivities of 65.2% for women and 71.4% for men, and specificities of 73.9% and 100%, respectively. These thresholds are close to those reported in another European study that included hospitalized patients (7 mm for women and 9 mm for men) [26], in comparison with BIA. An Asian study proposed higher thresholds in a healthier, younger community-dwelling population, also using BIA as the reference (10 mm for women and 11 mm for men), with higher sensitivity and specificity (90.9% et 92.2%), and based on the Asian Working Group for Sarcopenia (AWGS) criteria which are slightly more stringent than EWGSOP2, due to regional differences in body composition and lifestyle [25,27].

We also aimed to assess the prognostic value of rectus femoris thickness, ASMI as assessed by DXA, and the overall diagnosis of sarcopenia and severe sarcopenia according to EWGSOP2. In our cohort, none of these variables were significantly associated with in-hospital mortality nor at 1, 3 or 6 months post-discharge, although a trend was observed for rectus femoris thickness at 3 and 6 months. Sarcopenia and ASMI have previously been identified as predictors of mortality at 1, 2, 5, and 7 years in hip fracture patients when using DXA or BIA [5,9,28]. Combined quadriceps measurements have been linked to 2-year mortality in heart failure patients [29] and 3-year mortality in dialysis patients [30], suggesting that long-term follow-up may reveal the prognostic potential of POCUS, but to our knowledge, the prognostic value of rectus femoris thickness measured by ultrasound has not yet been evaluated in patients with hip fractures.

Rectus femoris thickness was also not predictive of severe postoperative complications, which is consistent with other studies in acute geriatric settings where only muscle echogenicity was associated with complications such as delirium, functional decline, incontinence, falls, pressure injuries, and infections [31]. Currently, echogenicity can only be measured using standard ultrasound and is not yet available on POCUS devices. Future technological advances, particularly in artificial intelligence, may make this possible. However, one study did find that POCUS measurements of rectus femoris thickness could predict falls and readmission within one month after discharge [32], suggesting a possible late prognostic role in the future.

Our study has several strengths. Patients were prospectively and systematically included, thus improving data reliability. By comparing a practical bedside tool with a reference method for frail older patients, new possibilities for muscle mass assessment are opened for frail, immobile patients. This technique is affordable, requires only a short training period, and shows excellent reproducibility.

There are limitations to the study. The small cohort size limited the statistical power, particularly for prognostic analyses and for conclusions regarding the diagnostic performance of rectus femoris thickness in men. This also limits the generalizability of our findings, especially in subgroup analyses. A longer follow-up would have better assessed the prognostic value of the muscle ultrasound. In addition, only 52 patients underwent both DXA and the ultrasound. This was due to ultrasound measurements starting a year later, which reduced overlap with earlier DXA assessments, and to DXA access being limited by logistical delays or the clinical condition of patients in the postoperative phase. Furthermore, the timing of handgrip strength measurement was not strictly standardized, as it depended on each patient’s postoperative status; this variability may have influenced the classification. Finally, although multivariate modeling would have been valuable to adjust for potential confounders, the low number of adverse events did not allow for robust analysis without risking overfitting. This limitation further underlines the need for larger validation studies.

However, our findings support the need to develop standardized protocols integrating POCUS into the assessment and monitoring of muscle health in older adults. Larger, long-term prospective studies are necessary to confirm its diagnostic and prognostic roles in the management of sarcopenia. POCUS may also have additional applications in orthogeriatric medicine, such as repeated measurements to monitor progress and adjusting rehabilitation intensity, as has been shown in intensive care unit settings [33,34]. Physical therapists could also use POCUS for real-time visual biofeedback to help patients visualize muscle changes and adapt their efforts to progress [35].

## 5. Conclusions

Our study showed that rectus femoris thickness measurement by POCUS ultrasound is a valid, rapid, and accessible alternative to DXA for diagnosing sarcopenia in geriatric patients hospitalized for hip fracture. We defined diagnostic thresholds as 5.7 mm for women and 9.3 mm for men. However, no clear prognostic value was observed for mortality or for severe postoperative complications. Further studies, conducted in larger cohorts with extended follow-up, will be necessary to confirm these preliminary results.

## Figures and Tables

**Table 1:** Descriptive data at admission.

Variable	*n*	*n* (%) Mean ± SD Median [IQR]
Socio-demographic, clinical, and geriatric data		
Age (years)	126	85.3 ± 6.6
Sex (Men)	126	35 (27.8)
Place of residence (living at home)	126	77 (61.1)
BMI (kg/m^2^)	126	23.0 [20.3–26.0]
Comorbidities	126	
Hypertension		86 (68.3)
Diabetes		25 (19.9)
Heart failure		46 (36.5)
Moderate to severe valvular disease		19 (15.1)
Chronic kidney disease		42 (33.3)
COPD		21 (16.7)
History of cerebrovascular disease		23 (18.3)
ADL (/24)	126	8 [7–15]
IADL (/8)	126	2 [0–5]
MNA-SF (/14)	126	7.8 ± 2.7
CIRS-G (/56)	126	16.4 ± 6.2
Cognitive impairment	126	55 (43.7)
Depression	126	36 (28.6)
Polypharmacy (>5 medications)	126	85 (67.5)
Surgical characteristics		
Type of hip fracture	126	
Intracapsular femoral neck fracture		65 (51.6)
Extracapsular intertrochanteric fracture		54 (42.8)
Extracapsular subtrochanteric fracture		7 (5.6)
Type of surgery	126	
Intramedullary nail		61 (48.4)
Hemiarthroplasty		52 (41.3)
Total hip replacement		7 (5.6)
Triple screw fixation		5 (3.9)
Dynamic hip screw		1 (0.8)
Time from admission to surgery (hours)	126	27 [15–50]
Duration of surgery (minutes)	126	84 [60–107]
Laboratory characteristics		
Hemoglobin (g/dL)	126	12.2 ± 2.0
White blood cell count (×10^3^/mm^3^)	126	10.7 [8.0–13.8]
C-reactive protein (mg/L)	126	11.0 [2.0–46.7]
Albumin (g/L)	126	36.0 [32.0–38.0]
25 hydroxy-Vitamin D (ng/mL)	126	26.0 [15.3–33.0]
Postoperative outcomes		
Severe complications (Clavien–Dindo score > 2)	126	9 (7.3)
Fall(s)	126	11 (8.7)
Delirium	126	24 (19.0)
Red blood cell transfusion	126	29 (23.0)
Pressure injury (injuries)	126	12 (9.5)
Length of hospital stay (days)	126	18 [16–23]
Mortality		
In-hospital		6 (4.8)
At 1 month		8 (6.3)
At 3 months		20 (16.0)
At 6 months		23 (19.5)

BMI: body mass index; COPD: chronic obstructive pulmonary disease; ADL: basic activities of daily living; IADL: instrumental activities of daily living; MNA-SF: Mini Nutritional Assessment—Short Form; CIRS-G: Cumulative Illness Rating Scale for Geriatrics.

**Table 2:** Sarcopenia-related data.

Variable	*n*	*n* (%) Mean ± SD Median [IQR]
Handgrip strength (kg)	123	12 [10–20]
Women	88	12 [8–16]
Men	35	23 [14–25]
ASMI (kg/m^2^)	103	5.5 [5.0–6.4]
Women	79	5.4 [4.9–6.2]
Men	24	6.3 [5.4–6.9]
Sarcopenia according to EWGSOP2	100	
No sarcopenia		27 (27)
Probable sarcopenia		30 (30)
Confirmed sarcopenia		28 (28)
Severe sarcopenia		15 (15)
Sarcopenia (binary variable)	100	43 (43)
Women	76	27 (35.5)
Men	24	16 (66.7)
Rectus femoris muscle thickness (mm)	69	9.4 ± 3.2
Women	51	9.2 ± 3.1
Men	18	10.2 ± 3.4

ASMI: Appendicular Skeletal Muscle Mass Index; EWGSOP2: European Working Group on Sarcopenia in Older People.

**Table 3:** Univariate logistic regression analyses of sarcopenia variables associated with the occurrence of severe complications (Clavien–Dindo Score > 2).

Variable	OR	CI 95%	*p*-Value
ASMI	0.80	[0.34–1.85]	0.598
Rectus femoris muscle thickness	1.11	[0.83–1.48]	0.483
Presence of sarcopenia (EWGSOP2)	0.80	[0.40–1.56]	0.508

OR: odds ratio; CI: confidence interval; ASMI: Appendicular Skeletal Muscle Mass Index; EWGSOP2: European Working Group on Sarcopenia in Older People.

**Table 4:** Univariate logistic regression analyses of sarcopenia variables associated with mortality.

Mortality	In-Hospital		1 Month		3 Months		6 Months	
N (%)	6 (4.8%)		8 (6.3%)		20 (16%)		23 (19.5%)	
	OR [95%]	*p*	OR [95%]	*p*	OR [95%]	*p*	OR [95%]	*p*
ASMI	0.07 [0.004–1.10]	0.058	0.66 [0.23–1.89]	0.436	0.84 [0.48–1.47]	0.540	1.14 [0.65–2.02]	0.641
RF thickness	0.78 [0.46–1.33]	0.361	0.86 [0.58–1.29]	0.471	0.81 [0.65–1.01]	0.062	0.82 [0.64–1.06]	0.126
Presence of sarcopenia (EWGSOP2)	NA	NA	NA	NA	0.36 [0.09–1.38]	0.135	0.44 [0.11–1.76]	0.248

OR: odds ratio; CI: confidence interval; ASMI: Appendicular Skeletal Muscle Mass Index; RF: rectus femoris; EWGSOP2: European Working Group on Sarcopenia in Older People; NA: not applicable, due to absence of deaths in the sarcopenic group.

## Data Availability

The original contributions presented in this study are included in the article. Further inquiries can be directed to the corresponding author. Raw data are available upon request.

## Supplementary Materials

The following supporting information can be downloaded at: https://www.mdpi.com/article/10.3390/jcm14155424/s1, Table S1: Univariate logistic regression analysis of variables associated with 6-month mortality; Table S2: Univariate logistic regression analysis of variables associated with the occurrence of severe complications.

## Abbreviations

The following abbreviations are used in this manuscript:

ADL	Activities of Daily Living
ASMI	Appendicular Skeletal Muscle Mass Index
AUC	Area Under the Curve
AWGS	Asian Working Group for Sarcopenia
BIA	Bioelectrical Impedance Analysis
BMI	Body Mass Index
CAM	Confusion Assessment Method
CI	Confidence Interval
CIRS-G	Cumulative Illness Rating Scale-Geriatric version
COPD	Chronic Obstructive Pulmonary Disease
CRP	C-reactive Protein
CT	Computed Tomography
DXA	Dual-energy X-ray Absorptiometry
EWGSOP	European Working Group on Sarcopenia in Older People
HGS	Handgrip Strength
IADL	Instrumental Activities of Daily Living
ICC	Intraclass Correlation Coefficient
MNA-SF	Mini Nutritional Assessment-Short Form
MRI	Magnetic Resonance Imaging
OR	Odds Ratio
POCUS	Point-of-Care Ultrasound
RF	Rectus Femoris
ROC	Receiver Operating Characteristics
SPPB	Short Physical Performance Battery
