# Supplementary material for: Diagnostic Value of Point-of-Care Ultrasound for Sarcopenia in Geriatric Patients Hospitalized for Hip Fracture
Source: J Clin Med. 2025 Aug 1;14(15):5424. doi: 10.3390/jcm14155424 (PMC12347811; doi:10.3390/jcm14155424)
Supplement: Supplementary file 1 [file jcm-14-05424-s001.zip › jcm-3752334-supplementary.pdf]

Supplementary Materials

Table S1: Univariate logistic regression analysis of variables associated with 6-month mortality.

Variable	OR	CI95%	p-value
Age	1,03	[0,95 – 1,12]	0,475
Male sex	3,13	[1,05 – 9,35]	0,040
Living in a nursing home	0,46	[0,16 – 1,35]	0,157
BMI	0,97	[0,86 – 1,10]	0,652
Hypertension	6,92	[0,87 – 54,86]	0,067
Diabetes	1,82	[0,56 – 5,86]	0,317
Heart failure	7,56	[2,24 – 25,50]	0,001
Moderate to severe valvular disease	0,90	[0,18 – 4,43]	0,898
COPD	1, 65	[0,47 – 5,76]	0,436
Chronic kidney disease	5,54	[1,76 – 17,45]	0,003
History of cerebrovascular disease	1,65	[0,47 – 5,76]	0,44
ADL	1,02	[0,92 – 1,13]	0,738
IADL	0,80	[0,63 – 1,00]	0,055
MNA-SF	0,84	[0,68 – 1,03]	0,101
CIRS-G	1,12	[1,02 – 1,22]	0,016
Cognitive impairment	1,93	[0,66 – 5,62]	0,229
Depression	1,96	[0,66 – 5,79]	0,224
Polypharmacy (>5 medications)	2,34	[0,62 – 8,82]	0,208
Time to surgery	1,00	[0,99 – 1,02]	0,562
Duration of surgery	1,00	[0,98 – 1,02]	0,887
Hemoglobin	0,92	[0,70 – 1,20]	0,526
CRP	1,01	[0,999 – 1,018]	0,083
Albumin	0,98	[0,86 – 1,12]	0,742
25-OH Vitamin D	1,06	[1,02 – 1,10]	0,006
Severe postoperative complications	30,67	[5,38 – 174,82]	<0,001
In-hospital fall recurrence	2,97	[0,68 – 12,87]	0,147
Delirium	1,56	[0,44 – 5,45]	0,490
Red blood cell transfusion	3,28	[1,06 – 10,14]	0,039
Pressure injuries	10,34	[2,41 – 44,35]	0,002
Length of hospital stay	1,03	[0,98 – 1,09]	0,245

OR: odds ratio; CI: confidence interval; BMI: body mass index; COPD: chronic obstructive pulmonary disease; ADL: activities of daily living; IADL: instrumental activities of daily living; MNA-SF: Mini Nutritional Assessment – Short Form; CIRS-G: Cumulative Illness Rating Scale For Geriatrics; CRP: C-reactive protein; 25-OH Vitamin D: 25-hydroxyvitamin D.

Table S2 : Univariate logistic regression analysis of variables associated with the occurrence of severe complications.

Variable	OR	CI95%	p-value
Age	1,02	[0,92 – 1,14]	0,667
Male sex	0,46	[0,12 – 1,83]	0,271
Living in a nursing home	0,79	[0,20 – 3,09]	0,730
BMI	0,86	[0,71 - 1,04]	0,111
Hypertension	3,95	[0,48– 32,72]	0,203
Diabetes	0,474	[0,06 – 3,98]	0,491
Heart failure	3,90	[0,92 - 16,43]	0,064
Moderate to severe valvular disease	5,33	[1,29 - 22,12]	0,021
COPD	2,69	[0,62 – 11,77]	0,188
Chronic kidney disease	4,39	[1,04 - 18,54]	0,044
History of cerebrovascular disease	2,37	[0,55 – 10,30]	0,248
ADL	0,93	[0,78 – 1,10]	0,381
IADL	0,94	[0,72 – 1,23]	0,643
MNA-SF	0,81	[0,61 – 1,09]	0,163
CIRS-G	1,03	[0,93 – 1,15]	0,582
Cognitive impairment	1,04	[0,27 – 4,08]	0,955
Depression	0,74	[0,15 – 3,76]	0,718
Polypharmacy (> 5 medications)	1,75	[0,35 – 8,83]	0,498
Time to surgery	1,01	[1,00 – 1,03]	0,046
Duration of surgery	1,01	[0,99 – 1,02]	0,642
Hemoglobin	1,07	[0,76 – 1,5]	0,703
CRP	1,02	[1,00– 1,02]	0,037
Albumin	0,89	[0,74 – 1,06]	0,197
25 OH Vitamin D	1,05	[0,99 – 1,10]	0,080
In-hospital fall recurrence	2,08	[0,22 – 19,60]	0,522
Delirium	1,46	[0,28 – 7,75]	0,657
Red blood cell transfusion	1,46	[0,28 – 7,75]	0,657
Pressure injuries	2,60	[0,55 – 16,42]	0,205
Length of hospital stay	1,06	[0,98 – 1,13]	0,097

OR: odds ratio; CI: confidence interval; BMI: body mass index; COPD: chronic obstructive pulmonary disease; ADL: activities of daily living; IADL: instrumental activities of daily living; MNA-SF: Mini Nutritional Assessment – Short Form; CIRS-G: Cumulative Illness Rating Scale for Geriatrics; CRP: C-reactive protein; 25-OH Vitamin D: 25-hydroxyvitamin D.
